# Disentangling environmental and spatial effects on phylogenetic structure of angiosperm tree communities in China

**DOI:** 10.1038/s41598-017-04679-5

**Published:** 2017-07-17

**Authors:** Hong Qian, Shengbin Chen, Jin-Long Zhang

**Affiliations:** 1 Research and Collections Center, Illinois State Museum, 1011 East Ash Street, Springfield, IL 62703 USA; 2 College of Environment, Chengdu University of Technology, Chengdu, 610059 China; 3 State Key Laboratory of Geohazard Prevention and Geoenvironment Protection, Chengdu University of Technology, Chengdu, 610059 China; 4 State Environmental Protection Key Laboratory of Synergetic Control and Joint Remediation for Soil & Water Pollution, Chengdu University of Technology, Chengdu, 610059 China; 5 Flora Conservation Department, Kadoorie Farm & Botanic Garden, Lam Kam Road, Tai Po, New Territories Hong Kong

## Abstract

Niche-based and neutrality-based theories are two major classes of theories explaining the assembly mechanisms of local communities. Both theories have been frequently used to explain species diversity and composition in local communities but their relative importance remains unclear. Here, we analyzed 57 assemblages of angiosperm trees in 0.1-ha forest plots across China to examine the effects of environmental heterogeneity (relevant to niche-based processes) and spatial contingency (relevant to neutrality-based processes) on phylogenetic structure of angiosperm tree assemblages distributed across a wide range of environment and space. Phylogenetic structure was quantified with six phylogenetic metrics (i.e., phylogenetic diversity, mean pairwise distance, mean nearest taxon distance, and the standardized effect sizes of these three metrics), which emphasize on different depths of evolutionary histories and account for different degrees of species richness effects. Our results showed that the variation in phylogenetic metrics explained independently by environmental variables was on average much greater than that explained independently by spatial structure, and the vast majority of the variation in phylogenetic metrics was explained by spatially structured environmental variables. We conclude that niche-based processes have played a more important role than neutrality-based processes in driving phylogenetic structure of angiosperm tree species in forest communities in China.

## Introduction

Species in a local community are a subset of those in the species pool of the region where the local community is located. Which species of the regional pool are assembled into the local community depends on the dispersal ability of species in the regional species pool to reach the local community and the degree to which the arrived species can tolerate abiotic and biotic conditions in the local community^1^. Two major classes of theories have been proposed to explain the assembly mechanisms of local communities. Niche-based theories suggest that deterministic processes (such as environmental filtering and species interactions) determine species composition of local communities^2, 3^, whereas neutrality-based theories suggest that stochastic processes (such as historical processes that influence the species pool and dispersal limitations) determine the assembly of local communities^4^. Over the past decades, there has been increasing evidence showing that both deterministic and stochastic processes have played roles in regulating species composition of local communities but the relative importance of these processes remains unclear, partly because these processes are not mutually exclusive and may shift in importance at different spatial scales or in different study contexts^1, 5–7^. The balance between deterministic and stochastic processes in governing species assemblages is influenced by prevailing environmental conditions^8^. Deterministic processes are expected to be strong drivers of community structure in harsh environments whereas stochastic processes may dominate in local communities with benign environmental conditions^8^.

On one hand, distributions of species along environmental gradients reflect such traits as cold or drought tolerance. Species–environment interactions are mediated by phenotypic traits, which have evolved to reflect adaptive tradeoffs^9, 10^. Increasing evidence has shown that some species traits are phylogenetically conserved^10, 11^. As a result, phylogenetically closely related species are expected to share more similar phenotypic traits with each other than with more distantly related species^12^. Because interactions between species and their environments are mediated by phenotypic tradeoffs^9, 10^, one would expect that the maintenance of ancestral traits in some phylogenetic clades and the emergence of derived traits in other phylogenetic clades limit the potential habitat types that can be occupied by different clades^13^. Processes of habitat filtering would likely exert a strong effect on species assembly and result in patterns of phylogenetic structure in communities. Because ancestral angiosperm clades originated in tropical environment^14^ and because closely related species are expected to have similar niches^15^, the phylogenetic niche conservatism hypothesis predicts that angiosperm species in community assemblages would be more phylogenetically related (clustered) in harsher (e.g., colder or dryer) environments, as commonly observed in empirical studies^16–18^.

On the other hand, spatial contingency can cause dispersal limitation of some clades across a region due to neutral dynamics or historical factors^19, 20^. Empirical studies have found that spatial effects are important in regulating phylogenetic structure^21^ because dispersal limitations alone can cause closely related species to occupy nearby sites, and environmental variables tend to have a strong spatial structure^22^.

The relative importance of niche-based deterministic processes (represented by climatic variation) and neutrality-based stochastic processes (represented by spatial variation) in generating phylogenetic community structure can be assessed by variation partitioning approaches^21^. Specifically, variation in phylogenetic structure can be partitioned into its purely environmental and spatial effects and their joint effects; this should allow one to assess the degree to which the environment and space each govern patterns of phylogenetic structure in community assemblages. If environmental filtering processes play a dominant role in structuring species assemblages, one would expect that the proportion of variation in community structure explained by environmental variables to be greater than that of spatial variables. Alternatively, if spatial contingency is a more important determinant of phylogenetic structure of the species assemblages, one would expect that the proportion of variation explained by spatial variables to be greater than that explained by environmental variables.

In this paper, we assess the relative importance of environmental heterogeneity and spatial contingency in driving phylogenetic structure of angiosperm tree assemblages in China, which covers a wide range of geographical extent and possesses a wide range of variation in climate (e.g., from tropical rain forests northward to boreal forests). Trees are large and thus tree stems and buds are easier to be damaged by cold climate, compared with shrubs and herbaceous plants that may be protected from cold temperature by understory vegetation and/or snow cover during winter; thus, trees are more sensitive to extreme climatic conditions than shrubs and herbs. Adaptation to cold tolerance may be difficult for tropical trees because it requires complex modifications of biochemistry, physiology and morphology to protect stems and buds from cold damage^13, 23^. Thus, angiosperm trees are an ideal taxonomic system for assessing the relative importance of environmental and spatial variables in shaping phylogenetic structure of community assemblages.

## Results

The number of angiosperm tree species per plot varied greatly among the forest plots examined, ranging from 2 to 66 with an average of 21 species per plot. Similarly, environment also varied greatly among the forest plots (Table S1). For example, annual mean temperature ranged from −4.7 to 23.2 °C, and annual precipitation ranged from 442 to 2032 mm (Table S1).

**Figure 1:**
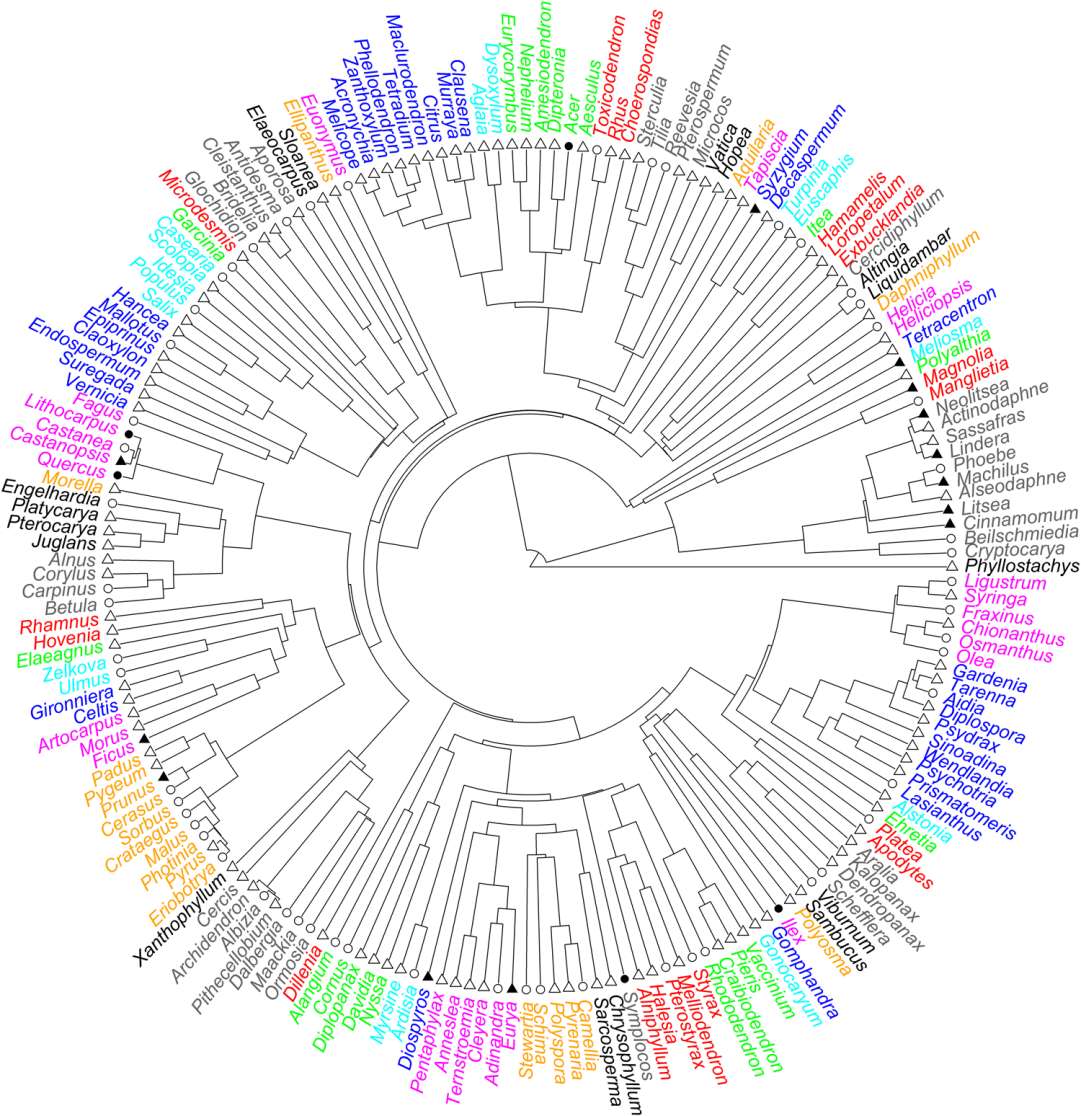
A phylogeny for 187 angiosperm tree genera in the studied forest plots (for illustrative purposes; analyses are based on a species-level phylogeny, see Methods). Genera within a cluster of the same color belong to the same family. The number of species in each genus was indicated by four different symbols: open triangles, 1 species; open circles, 2–5 species; filled triangles, 6–10 species; filled circles, > 10 species.

Phylogenetic structure of the forest communities varied across the spatial extent of the study at a varying degree, depending on the particular phylogenetic metric. PD was positively correlated with MPD and negatively correlated with MNTD (P < 0.05 in both cases; Fig. 1); there was no significant relationship between MPD and MNTD (Fig. 1). When the standardized effect sizes of these three metrics were considered, they were positively correlated to each other (P < 0.05 in all cases, Fig. 1).

PD, MPD, and MNTD each were significantly correlated with each of the eight environmental variables examined (P < 0.05 in all cases; Table 1). PD_ses_ was significantly correlated with seven of the eight environmental variables while MPD_ses_ and MNTD_ses_ were significantly correlated with only three and one environmental variables, respectively (Table 1). Compared with the three metrics of the standardized effect size, on average, their counterparts were more strongly correlated with environmental variables (Table 1; the mean of absolute values of the 24 correlation coefficients was 0.661 for PD, MPD and MNTD and 0.301 for PD_ses_, MPD_ses_ and MNTD_ses_; *t*-test, *P* < 0.001).

**Table 1:** Pearson’s correlation coefficients between phylogenetic metrics and environmental variables. Statistic significance: *P < 0.05, ^n^ *P* >  = 0.05.

Variable	PD	MPD	MNTD	PD_ses_	MPD_ses_	MNTD_ses_
BIO1	0.887^*^	0.824^*^	−0.476^*^	0.725^*^	0.290^*^	0.160^n^
BIO4	−0.864^*^	−0.778^*^	0.453^*^	−0.676^*^	−0.284^*^	−0.157^n^
BIO5	0.682^*^	0.706^*^	−0.394^*^	0.659^*^	0.197^n^	0.123^n^
BIO6	0.880^*^	0.819^*^	−0.468^*^	0.718^*^	0.284^*^	0.161^n^
BIO12	0.807^*^	0.729^*^	−0.647^*^	0.557^*^	0.044^n^	−0.122^n^
BIO15	−0.661^*^	−0.685^*^	0.582^*^	−0.542^*^	0.031^n^	0.133^n^
BIO17	0.616^*^	0.647^*^	−0.592^*^	0.468^*^	−0.100^n^	−0.233^n^
BIO18	0.571^*^	0.508^*^	−0.592^*^	0.228^n^	−0.047^n^	−0.295^*^

Of the six best-fit models for the relationships between phylogenetic metrics and environmental variables (Table 2), BIO6 (minimum temperature of the coldest month) was retained in five models and was the most important explanatory variable in four of the five models, as indicated by its standardized regression coefficients. Of the six phylogenetic metrics, environmental variables explained the highest amount (86.4%) of the variation in PD and the lowest amount (28.9%) of the variation in MNTD_ses_ (Table 2).

**Table 2:** Environmental variables retained in best-fit models and standardized regression coefficients (in parentheses). All models are significant (*P* < 0.05).

Variable	Predictors	*R* ^2^_adj_
PD	BIO1 (0.33), BIO12 (1.62), BIO17 (−0.89), BIO18 (−0.31)	0.864
MPD	BIO4 (0.95), BIO6 (1.75)	0.687
MNTD	BIO1 (−9.13), BIO5 (1.61), BIO6 (8.39), BIO15 (1.31)	0.520
PD_ses_	BIO1 (−13.71), BIO5 (2.83), BIO6 (12.35), BIO12 (1.66), BIO15 (0.73), BIO17 (−2.18)	0.502
MPD_ses_	BIO1 (−5.07), BIO5 (1.27), BIO6 (4.65), BIO12 (1.43), BIO17 (−1.14), BIO18 (−0.44)	0.619
MNTD_ses_	BIO4 (1.27), BIO6 (2.13), BIO17 (−0.82)	0.289

Spatial variables explained less amount of the variation in each phylogenetic metric, compared with environmental variables (compare Table 2 with Table 3). Of the six phylogenetic metrics, spatial variables explained the highest amount (85.8%) of the variation in PD and the lowest amount (14.8%) of the variation in MNTD_ses_ (Table 3).

**Table 3:** Spatial variables retained in best-fit models and standardized regression coefficients (in parentheses). All models are significant (*P* < 0.05).

Variable	Predictors	*R* ^2^_adj_
PD	PCNM1 (−0.65), PCNM4 (0.67), PCNM7 (−0.10)	0.858
MPD	PCNM1 (−0.72), PCNM2 (0.20), PCNM4 (0.29), PCNM7 (−0.14)	0.607
MNTD	PCNM1 (0.52), PCNM3 (0.10), PCNM4 (−0.23), PCNM6 (−0.02)	0.284
PD_ses_	PCNM3 (0.35), PCNM4 (0.30), PCNM6 (−0.26)	0.241
MPD_ses_	PCNM1 (−0.58), PCNM3 (0.16), PCNM4 (0.33), PCNM5 (−0.20), PCNM6 (−0.18)	0.509
MNTD_ses_	PCNM4 (0.37), PCNM6 (−0.20)	0.148

**Figure 2:**
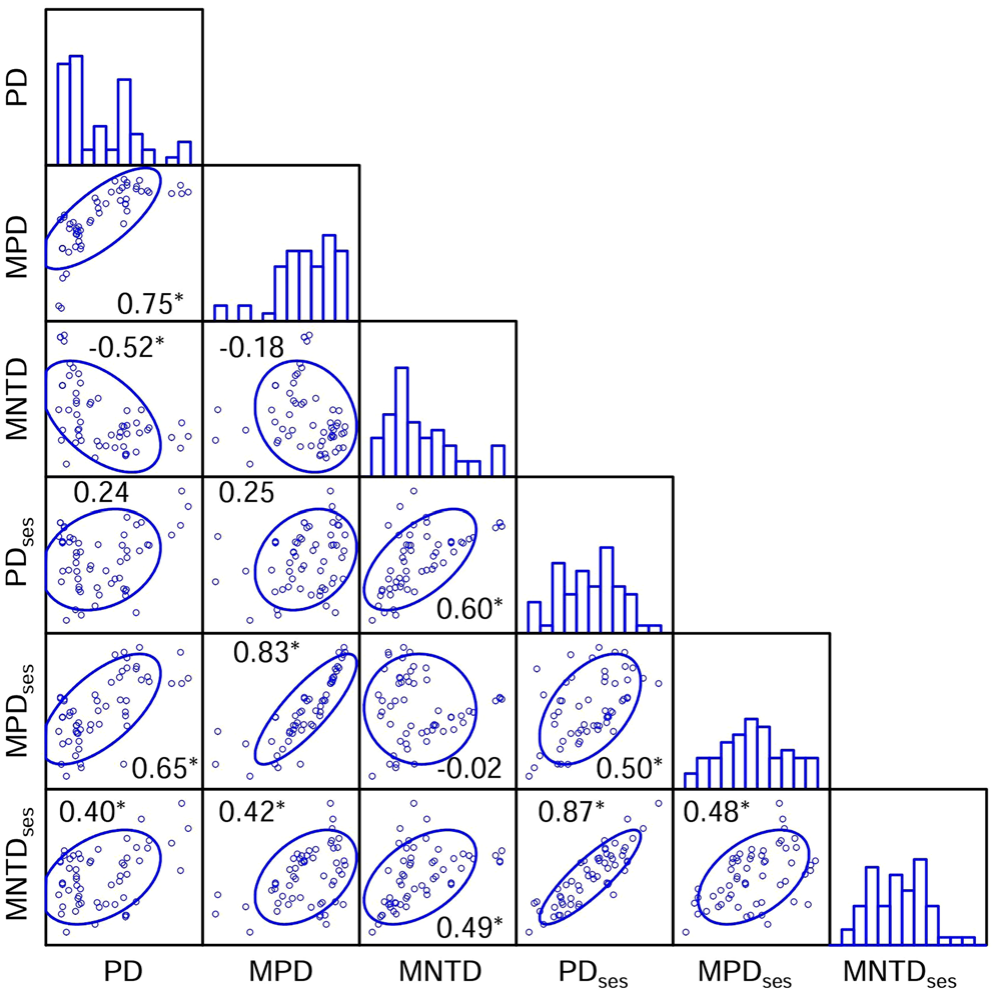
Histogram of each phylogenetic metric and relationships between phylogenetic metrics. An ellipse includes 75% of the sample. The center of each ellipse is the sample mean of the *x* and *y* variables in the plot, and its axes are determined by the standard deviations of *x* and *y*. Values are Pearson’s correlation coefficients (*Indicating *P* < 0.05).

When each phylogenetic metric was simultaneously regressed on the environmental and spatial variables retained in the two best-fit models for the phylogenetic metric (Tables 2 and 3), of the six phylogenetic metrics, environmental and spatial variables explained the highest amount (91.8%) of the variation in PD and the lowest amount (27.9%) of the variation in MNTD_ses_ (Fig. 2). Variation partitioning analyses showed that the variation in a phylogenetic metric explained independently by environmental variation was, on average, much larger than that explained independently by spatial structure (14% versus 4%; Fig. 2), and that of the variation in a phylogenetic metric that was explained by the environmental and spatial variables, the vast majority was explained by spatially structured environmental variation (i.e., the amount of the variation in a phylogenetic metric that was explained by the environmental and spatial variables jointly; Fig. 2). For example, environmental and spatial variables jointly explained 80.4% of the variation in PD (Fig. 2).

## Discussion

This study is one of the few that assess the relative importance of environmental and spatial factors in driving patterns of phylogenetic structure of species assemblages. Species assemblages examined here were distributed across wide spans in both environmental gradients and spatial extents (e.g., a long latitudinal gradient). These ample extents of environment and space are ideal for examining the relative importance of the two types of driving factors on the formation of phylogenetic structure.

Our study showed that among the eight environmental variables examined, mean annual temperature (BIO1) was most strongly correlated with phylogenetic structure (Table 1). However, when all environmental variables were simultaneously considered in a regression analysis, it is minimum temperature (BIO6) that was retained in the vast majority of the best-fit models. This suggests that minimum temperature is the most important driver of phylogenetic structure patterns for angiosperm tree species, at least in China. This is consistent with the climatic extreme hypothesis^23, 24^.

We found that MPD_ses_ was significantly and positively correlated with temperature, regardless of whether mean annual temperature or minimum temperature was considered. This indicates that angiosperm tree species in a forest community are phylogenetically more closely related with decreasing temperature. This pattern is consistent with the phylogenetic niche conservatism hypothesis, which predicts more phylogenetical clustering in colder environments. We also found that MNTD_ses_ was not correlated with any temperature variables (P > 0.05; Table 1). Because MPD_ses_ measures phylogenetic relatedness among taxa at both deep and shallow levels within a phylogenetic tree and emphasizes phylogenetic relatedness for major clades (e.g., orders and families) branching at deep nodes whereas MNTD_ses_ measures phylogenetic relatedness at a shallower level within the phylogenetic tree among taxa descending from superficial nodes, the fact that temperature is correlated with MPD_ses_ but not with MNTD_ses_ suggests that cold tolerance evolved at deep divergences in angiosperm evolutionary history.

Our variation partitioning analyses showed that the variation in phylogenetic metrics explained independently by environmental variation was much larger than that explained independently by spatial structure. Our environmental variables included only climate variables. If our environmental variables also included soil variables, which are not available for our forest plots, we believe environmental variables would have even stronger effects on phylogenetic metrics than did spatial variables, enhancing our conclusion. Our finding is consistent with that of Cisneros *et al*. (2016)^25^ who found that ∼12% and ∼1% of the variation in phylogenetic structure of bat communities in Costa Rica are independently explained by environmental and spatial variables, respectively. Duarte *et al*. (2012)^26^ also demonstrated that the phylogenetic structure of *Araucaria* forests in Brazil is better explained by environmental factors than by space. In a subtropical forest in China, Liu *et al*. (2013)^27^ also found that environmental variables explained more variation than did spatial variables for several tree traits (e.g. maximum height). Taken together, these findings suggest that niche-based processes (e.g., niche availability, species–habitat associations, and physiological limitations to environmental conditions) have played a more important role in driving phylogenetic structure patterns in local communities than neutrality-based processes (e.g., ecological drift, dispersal limitation, differential colonization or extinction dynamics). However, these findings are inconsistent with those of Gavilanez & Stevens (2013)^21^ who found that purely spatial processes play a stronger role in structuring primate communities than niche mechanisms in tropical America. More studies are needed to determine whether it is general that environment plays a more important role than space in driving phylogenetic structure of plant and animal communities.

Our results showed that the vast majority of the total variation in phylogenetic structure that was explained by environmental and spatial variables was explained jointly by the two types of variables, regardless of which phylogenetic metric was considered. For example, of 91.8% of the variation in PD that was explained by environmental and spatial variables, 80.4% was explained jointly by the two types of variables. Similar results have been found in other studies. For example, in Cisneros *et al*.’s (2016)^25^ study for bat communities in Costa Rica, on average, approximately 84% of the variation in phylogenetic structure is explained jointly by environmental and spatial variables. This overlapping effect by environment and space may represent a dispersal effect that is correlated with topography or the joint effect of multiple environmental factors that have a similar spatial structure (i.e., spatially structured environmental variation). It is difficult to tease apart the variation jointly explained by environment and space^28, 29^.

## Methods

### Study sites and data collection

**Figure 3:**
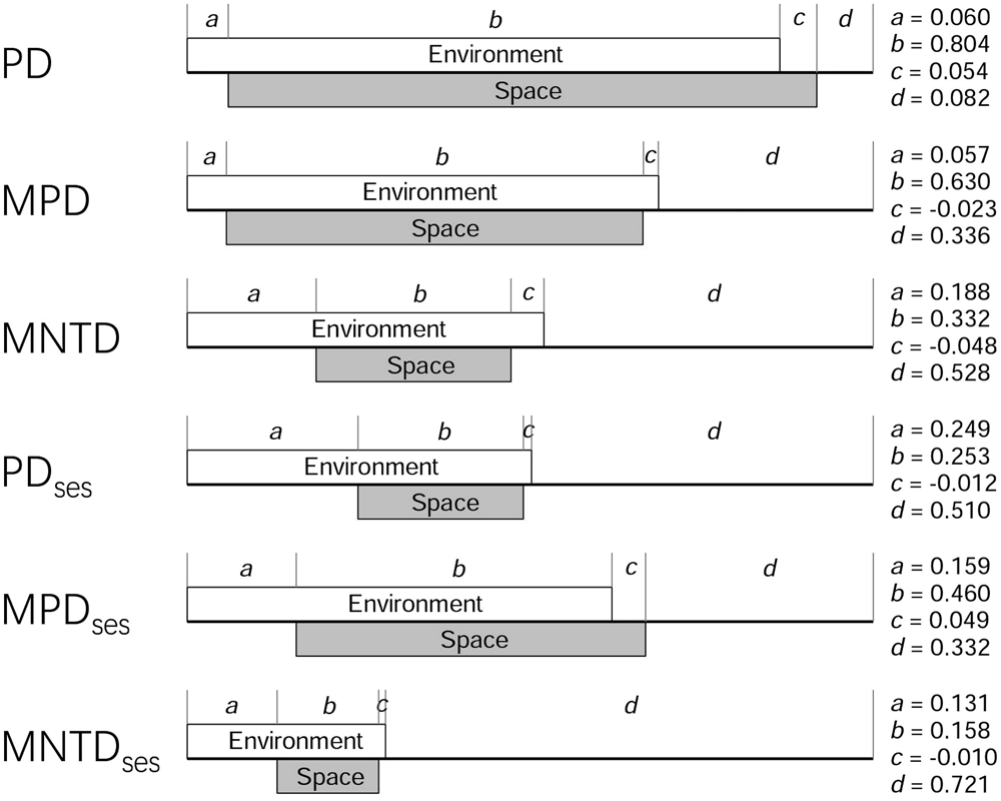
Results of partial regression analyses partitioning the variation in each phylogenetic metric into four portions: (**a**) uniquely accounted for by environmental variables, (**b**) accounted for jointly by environmental and spatial variables, (**c**) uniquely accounted for by spatial variables, and (**d**) unexplained variance. For each phylogenetic metric, environmental and spatial variables included in the partial regression analyses are shown in Tables 2 and 3.

Forest plots used in this study were sampled in 15 protected areas across China (see Fig. S1 in Qian *et al*. 2016^18^). These areas were distributed across a wide range of geographical space, covering a latitudinal gradient of 35° from tropical rain forests to boreal forests. We sampled four forest plots in each area. Each forest plot was 20 by 50 m (0.1 ha). Location (latitude, longitude, and elevation) of each plot was recorded. Woody individuals with diameter at breast height being 3 cm or larger were identified to species. All species included in this study are native tree species. Three forest plots each included less than two angiosperm tree species and were excluded because some phylogenetic metrics used in this study require at least two species in a forest plot. As a result, 57 forest plots were included in this study. A species list of angiosperm trees was compiled for each forest plot. Botanical nomenclature at the species level was standardized according to the Flora of China^30^. The 57 plots contained 462 angiosperm tree species in 187 genera and 63 families (Fig. 3).

### Phylogeny reconstruction

The phylogeny used in this study for the 462 species is the same phylogeny as in Qian *et al*. (2016)^18^. This phylogeny was generated using an updated version of Zanne *et al*.’s (2014)^31^ phylogeny (i.e., PhytoPhylo; Qian & Jin, 2016; available at https://github.com/jinyizju/S.PhyloMaker) as a backbone, which was in turn built based on seven gene regions (i.e., ribosomal cluster regions: 18 S rDNA, 26 S rDNA, ITS, chloroplast genes: *mat*K, *rbc*L, *atp*B, and the chloroplast *trn*L-F intron) and time-calibrated based on fossil data.

### Phylogenetic metrics

Faith’s phylogenetic diversity (PD) and Webb’s mean pairwise distance (MPD) and mean nearest taxon distance (MNTD) are commonly used metrics to quantify phylogenetic diversity^32–34^. Accordingly, we used them to quantify phylogenetic diversity in the angiosperm tree communities of this study. All these phylogenetic metrics are based on branch lengths of the phylogeny but they emphasize different depths of evolutionary histories across a phylogeny. PD measures the total amount of phylogenetic distance among species in a community. We used PD metric^32^ to quantify the phylogenetic diversity of each forest plot as the total phylogenetic branch length joining the basal node (i.e., the basal node of angiosperms in our case) to the tips of all the species in the forest plot. MPD measures the mean phylogenetic distance separating all assemblage members from each other (i.e., a tree-wide assessment of relatedness among co-occurring members) and thus quantifies the overall relatedness of the assemblage members. MNTD measures the average distance to the closest relative for each taxon (i.e., an assessment of terminal relatedness among co-occurring taxa). These three metrics are affected by species richness in community assemblages. To account for species richness, we calculated the standardized effect size (ses) of each phylogenetic diversity metric using the following formula: *X*_ses_ *= (X*_observed_ – *X*_randomized_ *)/SD*_randomized_, where *X*_ses_ represents the standardized effect size of a phylogenetic diversity metric (i.e., PD_ses_, MPD_ses_, or MNTD_ses_), *X*_observed_ represents the observed value of the metric, *X*_randomized_ represents the mean of randomized values, and *SD*_randomized_ represents the standard deviation of the randomized values. The null model that we used to calculate the metrics shuffled the names of taxa across the tips of the phylogeny 999 times. PD_ses_, MPD_ses_ and MNTD_ses_ are, respectively, PD, MPD and MNTD standardized with respect to a regional species pool; they reflect whether species in an assemblage are overdispersed or clustered with respect to the species pool. For each of PD_ses_, MPD_ses_ and MNTD_ses_, a positive value indicates phylogenetic evenness or overdispersion and a negative value indicates phylogenetic clustering. Note that MPD_ses_ and MNTD_ses_ are, respectively, equal to −1 NRI (net relatedness index) and −1 NTI (nearest taxon index)^34^. All phylogenetic metrics were calculated using Picante^35^.

### Environmental and spatial variables

We selected eight environmental variables to examine the relationship between environment and phylogenetic structure of the angiosperm tree communities. The environmental variables, which were extracted from the WorldClim database (http://www.worldclim.org)^36^, are annual mean temperature (BIO1), temperature seasonality (BIO4), maximum temperature of the warmest month (BIO5), minimum temperature of the coldest month (BIO6), annual precipitation (BIO12), precipitation seasonality (BIO15), precipitation of the driest quarter (BIO17), and precipitation of the warmest quarter (BIO18). Previous studies have shown that these environmental variables are among major determinants of animal and plant distributions^37^.

Spatial variables were represented by eigenvectors derived from principal coordinates of neighbour matrices (PCNM) based on the geographic coordinates of communities^22, 38^. PCNM allows assessing spatial effects on phylogenetic structure of local communities at multiple scales. PCNM variables were generated by performing a principal coordinate analysis (PCoA) on a truncated distance matrix connecting the forest plots. PCNM vectors were calculated with geographic distances between plots being truncated at the maximum distance connecting all sites (809.7 km), which was determined based on a minimum spanning tree criterion^39^. Seven PCNM variables resulted from the analysis for the forest plots. We used SAM 4.0 software^39^ to conduct the PCNM analysis.

### Data analysis

We conducted correlation analyses to assess the relationship of each of the six phylogenetic metrics with other five phylogenetic metrics and with the eight environmental variables. For each phylogenetic metric, we built models with all possible combinations of the eight environmental variables and used the corrected Akaike information criterion (AIC_c_) to evaluate performance of each model and to select the model with the lowest AIC_c_ as the best-fit model^40^. Similarly, we built models with all possible combinations of the seven spatial variables (PCNM vectors) and used AIC_c_ to select the best-fit model.

For each phylogenetic metric, we conducted a series of partial regressions^41^ to partition the variance of the phylogenetic metric. We first regressed each phylogenetic metric on the set of environmental variables and the set of spatial variables retained in the two best-fit models separately, and then regressed each phylogenetic metric on the two sets of variables simultaneously. We conducted variation partitioning analyses^41^ to partition the variation in each phylogenetic metric into four fractions: (1) variation accounted for by environmental variation alone, (2) variation accounted for by environmental and spatial variations jointly, (3) variation accounted for by spatial variation alone, and (4) variation accounted for by neither environmental nor spatial variation.

We used SYSTAT^42^ to conduct all statistical analyses.

## Electronic supplementary material

Supplementary Information
