# Supplementary material for: Disentangling environmental and spatial effects on phylogenetic structure of angiosperm tree communities in China
Source: Sci Rep. 2017 Jul 17;7:5634. doi: 10.1038/s41598-017-04679-5 (PMC5514081; doi:10.1038/s41598-017-04679-5)
Supplement: Supplementary file 1 — Supplementary Information [file 41598_2017_4679_MOESM1_ESM.pdf]

Disentangling environmental and spatial effects on phylogenetic structure of angiosperm tree communities in China

Hong Qian[1], Shengbin Chen[2], Jin-Long Zhang[3]

[1]Research and Collections Center, Illinois State Museum, 1011 East Ash Street, Springfield, IL 62703, USA. [2] College of Environment and Civil Engineering, Chengdu University of Technology, Chengdu, 610059, China. [3]Flora Conservation Department, Kadoorie Farm & Botanic Garden, Lam Kam Road, Tai Po, New Territories, Hong Kong. Correspondence and requests for materials should be addressed to S.C. (email: chainpin@126.com).

Supplementary Information

Table S1. Descriptive statistics of environmental variables.

Variable	Minimum	Maximum	Mean
BIO1	-4.7	23.2	9.1
BIO4	3.2	16.9	9.9
BIO5	22.4	32.2	26.9
BIO6	-36.6	13.9	-11.1
BIO12	442.0	2032.0	1132.0
BIO15	47.0	105.0	79.8
BIO17	13.0	189.0	76.4
BIO18	282.0	715.0	495.8

Variable full name: annual mean temperature (BIO1), temperature seasonality (BIO4), maximum temperature of the warmest month (BIO5), minimum temperature of the coldest month (BIO6), annual precipitation (BIO12), precipitation seasonality (BIO15), precipitation of the driest quarter (BIO17), and precipitation of the warmest quarter (BIO18). The temperature data are in °C and the precipitation data are in mm.
